# Tackling the unyielding: testing two novel approaches against NDM-producing Enterobacterales and *Pseudomonas aeruginosa* isolates collected in India

**DOI:** 10.1128/spectrum.00497-24

**Published:** 2024-11-19

**Authors:** Yamuna Devi Bakthavatchalam, Bijayini Behera, Anand Shah, Purva Mathur, Raja Ray, Bashir Ahmed Fomda, Kamini Walia, Balaji Veeraraghavan

**Affiliations:** 1 Department of Clinical Microbiology, Christian Medical College, Vellore, Tamil Nadu, India; 2 Department of Microbiology, All India Institute of Medical Sciences, Bhubaneswar, Odisha, India; 3 Department of Microbiology, Zydus Hospitals, Ahmedabad, Gujarat, India; 4 Department of Clinical Microbiology, All India Institute of Medical Sciences, New Delhi, India; 5 Department of Microbiology, Institute of Post Graduate Medical Education and Research, Kolkata, India; 6 Department of Microbiology, Sher-i-Kashmir Institute of Medical Sciences, Srinagar, Jammu and Kashmir, India; 7 Division of Epidemiology and Communicable Diseases, Indian Council of Medical Research, New Delhi, India; Nevada State Public Health Laboratory, Reno, Nevada, USA

**Keywords:** cefiderocol, cefepime/zidebactam, NDM, India

## Abstract

The *in vitro* activity of two novel antibiotics with different modes of action, (i) siderophore cefiderocol and (ii) β-lactam-enhancer mechanism-based cefepime/zidebactam, was tested against New Delhi Metallo-β-lactamase (NDM)-producing Enterobacterales and *Pseudomonas aeruginosa* collected in India. Minimum inhibitory concentrations of antibiotics against multicentric NDM-producing *Escherichia coli* (*n* = 117), *Klebsiella pneumoniae* (*n* = 103), and *P. aeruginosa* (*n* = 72) were determined by the reference broth microdilution method. Among *E. coli*, 111 isolates were NDM-alone, and six were NDM + OXA-48-like producers. Among *K. pneumoniae*, 47 and 56 isolates were NDM-alone and NDM + OXA-48-like producers, respectively. All *E. coli* isolates harbored four amino acid inserts in their penicillin-binding protein 3. Using the highest susceptible breakpoint among CLSI, FDA, and EUCAST interpretive criteria, cefiderocol susceptibility was 39.3%, ≤80%, and 57%, for NDM ± OXA-48-like-producing *E. coli*, NDM ± OXA-48-like-producing *K. pneumoniae,* and NDM-producing *P. aeruginosa*, respectively. At a cefepime break point of ≤8 mg/L, 100% of Enterobacterales and ≥90% of *P. aeruginosa* isolates were cefepime/zidebactam-susceptible. NDM being a dominant carbapenemase among Enterobacterales and *P. aeruginosa* in India, the variable activity of cefiderocol against NDM producers is a concern. Post approval, cefepime/zidebactam could offer a promising treatment option against NDM producers.

**IMPORTANCE:**

Metallo-β-lactamases are therapeutically challenging due to the limited treatment options. Against such isolates, currently approved newer β-lactam/β-lactamase inhibitor combinations are ineffective. In this study, we tested siderophore cephalosporin, cefiderocol, which utilizes an unconventional iron uptake pathway for efficient cellular penetration, and cefepime/zidebactam that utilizes novel β-lactam enhancer mechanisms for overcoming diverse carbapenemases. Cefiderocol showed limited activity against *Escherichia coli* isolates co-harboring New Delhi metallo-β-lactamase (NDM) with PBP3 insert, dual carbapenemase (NDM with OXA-48 like)-producing *Klebsiella pneumoniae*, and NDM-producing *Pseudomonas aeruginosa* isolates, while cefepime/zidebactam potently inhibited NDM-producing Enterobacterales and *P. aeruginosa* isolates. NDM being a dominant carbapenemase among Enterobacterales and *P. aeruginosa* in India, the variable activity of cefiderocol against NDM producers is a concern. Post approval, cefepime/zidebactam could offer a promising treatment option against NDM producers.

## OBSERVATION

Metallo-β-lactamases (MBLs) are therapeutically the most problematic carbapenemases as they exhibit a broad substrate profile, have a higher degree of hydrolytic activity, and are not inhibited by any of the marketed β-lactamase inhibitors (1, 2). Among the various types of MBLs, in Asia and particularly in India, New Delhi Metallo-β-lactamases (NDMs) are the most common carbapenem resistance mechanism both in Enterobacterales and in *Pseudomonas aeruginosa* (3). Studies show that about 90% of carbapenem-resistant *Escherichia coli* and about 60% of carbapenem-resistant *Klebsiella pneumoniae* in India harbor NDMs (4). The therapeutic challenge is further enhanced by the fact that most NDM-producing *E. coli* also harbor four amino acid inserts in their penicillin-binding protein (PBP) 3 that impacts the binding of sole PBP3-targeting β-lactams such as cefiderocol and aztreonam (5, 6). Further compounding the problem, in India, >90% of NDM-producing *K. pneumoniae* co-produce OXA-48-like carbapenemases, thereby further curtailing the treatment options (7). Against such isolates, currently approved newer β-lactam/β-lactamase inhibitor combinations (ceftazidime/avibactam, imipenem/relebactam, and meropenem/vaborbactam) are ineffective as the β-lactamase inhibitors in these combinations are unable to neutralize MBLs (8). Moreover, relebactam and vaborbactam do not inhibit OXA-48-like enzymes. In the backdrop of such challenging epidemiology, we tested siderophore cephalosporin, cefiderocol, which utilizes an unconventional iron uptake pathway for efficient cellular penetration, and cefepime/zidebactam that utilizes novel β-lactam enhancer mechanisms for overcoming diverse carbapenemases (9–11). Currently, cefepime/zidebactam (WCK 5222) is undergoing a registrational global Phase 3 trial (ClinicalTrials.gov ID: NCT04979806), while cefiderocol is already approved in the United States and Europe and likely to be marketed in India. While there are several studies that tested cefiderocol or cefepime/zidebactam against NDM producers, such studies did not involve isolates collected in India, a region with unusual combinations of carbapenem-impacting resistance mechanisms. In this context, we undertook a study to determine the minimum inhibitory concentrations (MICs) of cefiderocol and cefepime/zidebactam against a panel of NDM-producing Enterobacterales and *P. aeruginosa* collected in India.

The study isolates (non-duplicate) were picked from a freezer stock of blood culture-positive, carbapenem-resistant organisms, which were identified (during 2022) in the routine susceptibility testing. All the blood specimens were from hospitalized patients. Bacterial species were confirmed at a central laboratory (Christian Medical College, Vellore, India) using MALDI-TOF-based VITEK MS (bioMérieux). The isolates were collected from six tertiary-care hospitals across India during 2021–2022. The panel comprised *E. coli* (*n* = 117), *K. pneumoniae* (*n* = 103), and *P. aeruginosa* (*n* = 72), which were positive for *bla*_NDM_ in the polymerase chain reaction (PCR). Among *E. coli*, 111 isolates were positive for *bla*_NDM_ alone, while six were positive for both *bla*_NDM_ and *bla*_OXA-48-like_. Similarly, among *K. pneumoniae*, 47 isolates were positive for *bla*_NDM_ alone, and 56 were positive for both *bla*_NDM_ and *bla*_OXA-48-like_ (12). MICs of cefiderocol were determined using the iron-depleted, cation-adjusted Mueller Hinton broth (Becton Dickinson, BD BBL, New Jersey, USA) as recommended by the Clinical and Laboratory Standards Institute (CLSI) (13). Comparator antibiotics, ceftazidime/avibactam, and meropenem were tested against all the isolates, while aztreonam/avibactam was tested only against *E. coli* and *K. pneumoniae*. MICs of cefepime/zidebactam and other comparators were determined by broth microdilution according to the method approved by CLSI (13). Ceftazidime, cefepime, and meropenem were procured from the Sigma-Aldrich Company (St. Louis, MO, USA), and cefiderocol, zidebactam, and avibactam were obtained from MedKoo Biosciences, Inc. (Morrisville, NC, USA). For each studied drug, respective CLSI-recommended QC strains were included to ensure the accurate determination of MICs. These include *E. coli* ATCC 25922, *K. pneumoniae* ATCC 700603, *K. pneumoniae* ATCC BAA-1705, *P. aeruginosa* ATCC 27853, and *Acinetobacter baumannii* NCTC13304. Cefepime/zidebactam MICs were determined in 1:1 ratio. A fixed 4-mg/L concentration of avibactam was employed to determine the MICs of aztreonam/avibactam and ceftazidime/avibactam. Susceptibilities of isolates to cefiderocol and the comparator antibiotics were interpreted by CLSI (13), FDA, and EUCAST (v 14.0) criteria, while for cefepime/zidebactam, breakpoints of cefepime as well as reported provisional break points were considered (14–16). For aztreonam/avibactam, a recently published EUCAST break point was considered (EUCAST website communication dated 22 May 2024). All the NDM-producing *E. coli* isolates (*n* = 117) were found to harbor four amino acid inserts (YRIN or YRIK) in their PBP3 as determined by a previously published PCR method (8).

Table 1 shows the MIC distribution of cefiderocol, cefepime/zidebactam, and the comparator antibiotics. Since defining the activity trend of cefiderocol against the given isolate population is challenging due to the differences in the CLSI, FDA, and EUCAST interpretive criteria, for each subset of isolates, we compared the activity of cefiderocol, and preference was given to the highest susceptible break point from the three criteria.

**TABLE 1:** MIC distribution of cefiderocol and comparator antibiotics against NDM-producing Enterobacterales and *P. aeruginosa* collected in India^*a*^

Organism group (*n*)/antibiotic	Number of isolates with MIC (mg/L) [% cumulative inhibition]									% susceptible			
	≤0.12	0.25	0.5	1	2	4	8	16	≥32	Provisional^*b*^	CLSI	FDA	EUCAST
NDM ± OXA-48-like-producing *E. coli* (*n* = 117)**^*c*^**													
Cefiderocol					6 [5.1]	40 [39.3]	29 [64.1]	10 [72.6]	32 [100]	–^*d*^	39.3	39.3	5.1
Cefepime/zidebactam	76 [65]	22 [83.8]	15 [96.6]	4 [100]						100	100	100	100
Aztreonam/avibactam	5 [4.3]	6 [9.4]	6 [14.5]	12 [24.8]	23 [44.4]	32 [71.8]	21 [89.7]	10 [98.3]	2 [100]	–	–	–	71.8
Ceftazidime/avibactam								3 [2.4]	114 [100]	–	0	0	0
Meropenem								5 [4.3]	112 [100]	–-	0	0	0
NDM-producing *K. pneumoniae* (*n* = 47)													
Cefiderocol			1 [2.1]	10 [23.4]	17 [59.6]	9 [78.7]	7 [93.6]	3 [100]		–	78.7	78.7	59.6
Cefepime/zidebactam	3 [6.4]	9 [25.5]	12 [51.1]	11 [74.5]	5 [85.1]	5 [95.7]	2 [100]			100	100	100	74.5
Aztreonam/avibactam	32 [68.1]	11 [91.5]	2 [95.7]	0 [95.7]	1 [97.9]	1 [100]				–	–	–	100
Ceftazidime/avibactam									47 [100]	–	0	0	0
Meropenem								1 [2.1]	46 [100]	–	0	0	0
NDM + OXA-48-like-producing *K. pneumoniae* (*n* = 56)													
Cefiderocol			1 [1.8]	8 [16.1]	17 [46.4]	19 [80.4]	4 [87.5]	1 [89.3]	6 [100]	–	80.4	80.4	46.4
Cefepime/zidebactam	5 [8.9]	1 [10.7]	9 [26.8]	16 [55.4]	18 [87.5]	7 [100]				100	100	100	55.4
Aztreonam/avibactam	11 [19.6]	34 [80.4]	9 [96.4]	0 [96.4]	0 [96.4]	0 [96.4]	2 [100]			–	–	–	96.4
Ceftazidime/avibactam								1 [1.8]	55 [100]	–	0	0	0
Meropenem								1 [1.8]	55 [100]	–	0	0	0
NDM-producing *P. aeruginosa* (*n* = 72)													
Cefiderocol		1 [1.4]	2 [4.1]	2 [6.9]	14 [26.4]	22 [56.9]	14 [76.4]	10 [90.3]	7 [100]	–	56.9	6.9	26.4
Cefepime/zidebactam					4 [5.6]	20 [33.3]	41 [90.3]	7 [100]		100	90.3	90.3	100
Meropenem								4 [5.6]	68 [100]	–	0	0	0

^*a*^ The gray-shaded boxes indicate MIC_50_ and MC_90_ values of the antibiotics.

^*b*^ Susceptibilities to cefepime/zidebactam are reported based on interpretive criteria for standalone cefepime as per CLSI or FDA or EUCAST, as well as based on reported provisional susceptibility break points; ≤8 mg/L for Enterobacterales and ≤32 mg/L for *P. aeruginosa* based on the anticipated clinical dose of cefepime/zidebactam (2 g cefepime + 1 g zidebactam administered every 8 h) despite PK/PD data supporting a cefepime/zidebactam-susceptible MIC break point of ≤64 mg/L (14–16).

^*c*^ Among 117 NDM-producing *E. coli*, six isolates also produced OXA-48-like carbapenemase.

^*d*^ –, not applicable.

Against NDM ± OXA-48-like-producing *E. coli*, regardless of the criteria applied, cefiderocol showed limited activity. Even after the highest susceptible break point of ≤4 mg/L (CLSI and FDA criteria) was applied, only 39.3% of the isolates were within the coverage of cefiderocol, while applying EUCAST breakpoint, the susceptibility dropped to 5.1%. With respect to cefepime/zidebactam, the combination was highly potent with all the isolates inhibited at 1 mg/L, way below its provisional break point of ≤8 mg/L applicable to Enterobacterales. The susceptibility rate to aztreonam/avibactam (applying EUCAST criterion) was 71.8%, thus showing better activity compared to that of cefiderocol.

As compared to its activity against the subset of *E. coli* isolates, cefiderocol exhibited an improved activity against NDM ± OXA-48-like-producing *K. pneumoniae*. At the susceptible break point of ≤4 mg/L, cefiderocol inhibited 78.7% and 80.4% of tested NDM or NDM + OXA-48-like-producing *K. pneumoniae* isolates, respectively. However, using EUCAST criteria, these susceptibilities fell to 46.4% to 59.6%. At ≤8 mg/L (cefepime-susceptible dose-dependent break point), cefepime/zidebactam inhibited all the isolates albeit MIC distribution was wider compared to that of *E. coli*. At aztreonam/avibactam’s susceptible break point of ≤4 mg/L, >90% of NDM or NDM + OXA-48-like-producing *K. pneumoniae* were inhibited.

Against NDM-producing *P. aeruginosa*, cefiderocol MICs were widespread with 56.9% of isolates inhibiting ≤4 mg/L (CLSI criteria), while using FDA and EUCAST criteria, the susceptibilities were 6.9% and 26.4%, respectively. Against the same set of isolates, cefepime/zidebactam inhibited 90.3% of isolates at cefepime’s susceptible break point of ≤8 mg/L, while at its PK/PD break point of ≤32 mg/L, 100% of isolates were susceptible. Aztreonam/avibactam was not tested against NDM-producing *P. aeruginosa* as previous studies have shown that only minor proportions of such isolates show susceptibility to this combination (17, 18).

In the present study, the activity of cefiderocol against the NDM producers was lower compared to its reported activity against isolates producing other MBLs (IMP and VIM) or serine carbapenemases (KPC and OXA-48-like) (19). These results are in agreement with previous studies that tested cefiderocol against similar isolates collected from other parts of the world. For instance, a report from China CRE-Network showed that out of 1,158 carbapenem-resistant Enterobacterales collected in China, 30 isolates were resistant to cefiderocol, and among them, 26 isolates were *E. coli* which invariably harbored *bla*_NDM_ and four amino acid inserts in PBP3 (6). A recent report from Saudi Arabia also showed the limited activity of cefiderocol against NDM-producing *E. coli* and *K. pneumoniae* (28.6% to 43% susceptibility at ≤4 mg/L) although the number of study isolates was lower (20). With respect to *P. aeruginosa*, previous publications describing the activity of cefiderocol against NDM-producing isolates are scarce.

The vulnerability of cefiderocol to NDM producers seems to be due to two resistance mechanisms: the hydrolytic activity of NDM on cefiderocol and the presence of four amino acid inserts in the PBP3 (only in *E. coli*). This proposition is supported by a previously reported improvement in the activity of cefiderocol against NDM producers when combined with dipicolinic acid, an MBL inhibitor, yet 20/40 isolates continued to show a protected cefiderocol MIC of ≥1 mg/L, surpassing the MICs against wild types (21). Another study showed several fold increases in cefiderocol MICs against *E. coli* or *P. aeruginosa* cloned with *bla*_NDM_ variants, while little or no increase in MICs was observed when cloned with *bla*_VIM_, *bla*_KPC-2_, and *bla*_oxa-48_ variants (22). Given that PBP3 is the only target of cefiderocol, it is conceivable that modification in PBP3 through four amino acid insertion results in reduced target-level affinity, which in conjunction with NDM-mediated hydrolysis results in loss of cefiderocol potency (6).

Our finding from this study is a concern, as India has been witnessing a dominance of NDM among Enterobacterales and *P. aeruginosa* and clinicians are desperately looking for safer and effective substitutes for polymyxins that are currently considered as salvage therapies for unyielding infections caused by such pathogens. This study indicates that cefiderocol may not be able to comprehensively address the challenge of NDMs and there is a continued need for novel options with assuring coverage of NDM-producing Enterobacterales and *P. aeruginosa*.

In our study, we also tested two Phase 3 stage antibiotics, cefepime/zidebactam (for Enterobacterales and *P. aeruginosa*) and aztreonam/avibactam (only for Enterobacterales), and their activity pattern is consistent with previous reports (8, 18). The antibacterial activity of cefepime/zidebactam against MBL producers is mediated through concurrent inactivation of both PBP3 and PBP2 by cefepime and zidebactam, respectively, termed as β-lactam enhancer action (10, 23). For cefepime/zidebactam, at ≤8 mg/L, 100% susceptibility was observed for Enterobacterales regardless of resistance mechanism, while >90% susceptibility was observed for *P. aeruginosa*. At cefepime/zidebactam PK/PD break point of 32 mg/L, the susceptibility rose to 100% for *P. aeruginosa*, indicating that this combination holds promise for the infections caused by MBL (including NDM)-producing Enterobacterales and *P. aeruginosa* (14). Importantly, cefepime/zidebactam activity was highly consistent for NDM-producing *E. coli* concurrently harboring four amino acid inserts in PBP3 (5, 8). In contrast, similar to cefiderocol, the activity of aztreonam/avibactam was adversely impacted by the presence of four amino acid inserts in PBP3 of NDM-producing *E. coli*, though the latter maintains potent activity against NDM-producing *K. pneumoniae* (8).

One of the limitations of this study is the lack of information on NDM variants; however, past studies employing Enterobacterales isolates collected in India have reported that NDM-5 and NDM-2 exclusively dominate the molecular epidemiology among NDMs. For instance, in one of the studies, among overall NDM-producing *Klebsiella* (*n* = 124), the NDM-5 and NDM-1 constituted 73% and 24% of the tested isolates, respectively, while NDM-7 (one isolate) and NDM-9 (three isolates) were relatively infrequent (7). Another limitation of the study emanates from the reported concerns regarding the reproducibility and accuracy of MICs for cefiderocol that potentially could also influence our results. However, being cognizant of this issue, we ensured that the MICs of cefiderocol were within the CLSI-specified QC range in each MIC run.

To sum up, our study highlights that, in India, NDM and PBP3 inserts are emerging as key determinants driving resistance to cefiderocol in Enterobacterales and *P. aeruginosa*. Therefore, it is vital that large-scale surveillance studies are conducted on an on-going basis to delineate the therapeutic role of cefiderocol in India. Upon its successful clinical development, cefepime/zidebactam, which utilizes a β-lactam enhancer action, is a promising treatment option for infections caused by NDM-producing Enterobacterales and *P. aeruginosa*.

## Data Availability

The data sets generated during the current study are available from the corresponding author on reasonable request.
